# Variable blocking temperature of a porous silicon/Fe_3_O_4_ composite due to different interactions of the magnetic nanoparticles

**DOI:** 10.1186/1556-276X-7-445

**Published:** 2012-08-08

**Authors:** Klemens Rumpf, Petra Granitzer, Puerto M Morales, Peter Poelt, Michael Reissner

**Affiliations:** 1 Institute of Physics, Karl Franzens University Graz, Universitaetsplatz 5, Graz, A-8010, Austria; 2 Instituto de Ciencia de Materials de Madrid, CSIC, Cantoblanco, Madrid, 28049, Spain; 3 Institute for Electron Microscopy, University of Technology Graz, Steyrergasse 17, Graz, A-8010, Austria; 4 Institute of Solid State Physics, Vienna University of Technology, Wiedner Hauptstr. 8, Vienna, A-1040, Austria

**Keywords:** porous silicon, iron oxide nanoparticles, superparamagnetism, 68.65.-k, 75.75.-c, 81.16.-c

## Abstract

In the frame of this work, the aim was to create a superparamagnetic nanocomposite system with a maximized magnetic moment when magnetized by an external field and a blocking temperature far below room temperature. For this purpose, iron oxide nanoparticles of 3.8-, 5- and 8-nm size have been infiltrated into the pores of porous silicon. To fabricate tailored magnetic properties of the system, the particle size and the magnetic interactions among the particles play a crucial role. Different concentrations of the particles dispersed in hexane have been used for the infiltration to vary the blocking temperature *T*_B_, which indicates the transition between the superparamagnetic behavior and blocked state. *T*_B_ is not only dependent on the particle size but also on the magnetic interactions between them, which can be varied by the particle-particle distance. Thus, a modification of the pore loading on the one hand and of the porous silicon morphology on the other hand results in a composite material with a desired blocking temperature. Because both materials, the mesoporous silicon matrices as well as the Fe_3_O_4_ nanoparticles, offer low toxicity, the system is a promising candidate for biomedical applications.

## Background

In recent years, magnetic nanoparticles attracted high attention in various fields of nanoscience. Not only is the change of the physical properties of low-dimensional materials compared to their bulk materials of interest, but their applicability in various fields is also a developing subject. Magnetic materials in the nanoscale range are utilized in magnetic data storage [1] and GMR devices [2], and also in biological and medical applications, magnetic particles are employed [3]. The magnetic properties of magnetite nanoparticles embedded in a nonmagnetic matrix have been figured out, which show the dependence of the magnetic behavior on the utilized matrix material [4]. Furthermore, magnetite nanoparticles are extensively used for potential biomedical applications such as imaging of diseases (e.g., cancer and diabetes) or in cellular therapy [5]. One precondition of magnetic particles for the utilization in biomedicine is the vanishing magnetic remanence when the external magnetic field is switched off, which requires that particles be superparamagnetic and do not magnetically interact. Thus, the blocking temperature should exhibit low values, but in any case, it has to be far below room temperature. Porous silicon, a versatile material which is also biodegradable, can be used as a matrix. Recently, works have been carried out, for example, by the groups of Sailor and Ferrari, concerning a combination of porous silicon and iron oxide nanoparticles applied in biomedicine [6,7]. In the following work, the optimization of the magnetic properties, meaning the magnetic moment, to be as high as possible and the transition temperature between superparamagnetic and ferromagnetic-like behaviors of the porous silicon/iron oxide nanocomposites to be sufficiently low will be figured out to show that the system is applicable for biomedicine.

## Methods

**Figure 1:**
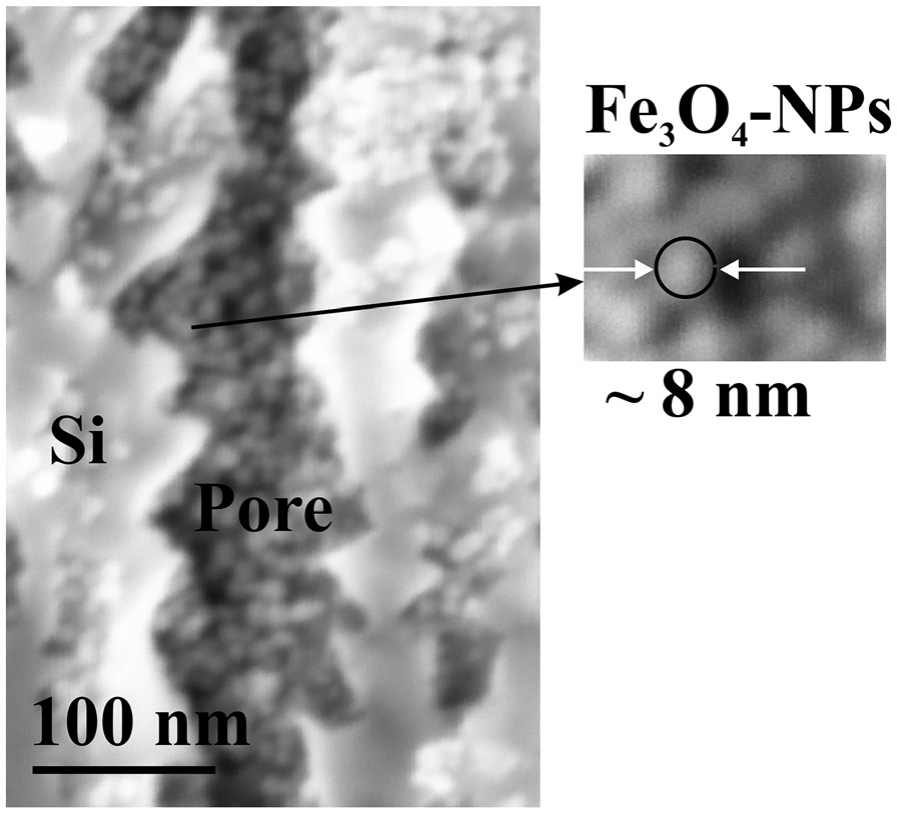
**Magnetite nanoparticles with an 8-nm average size infiltrated into the pores of porous silicon.** The distance between the particles is near 4 nm (twice the thickness of the coating). The concentration of the particle solution was 8 mg Fe/ml. The image shows a cross-sectional region near the pore tips.

Two different types of porous silicon matrices in the mesoporous regime with typical pore diameters around 50 and 90 nm have been fabricated by anodization using highly (100) *n*-doped silicon (10^19^/cm^3^) as substrate [8]. By keeping the current density constant at 100 and 115 mA/cm^2^, respectively, a quasi-regular arrangement of pores with a mean distance of 50 nm (pore diameter 50 nm) and 35 nm (pore diameter 90 nm) between them could be achieved. By increasing the current density from 50 to 125 mA/cm^2^, the pore diameter increases from about 25 to 100 nm, and concomitantly, the thickness of the pore walls decreases from 60 to 40 nm. The pore formation process is described in detail in a previous publication [9]. The samples have been etched for 8 min to reach a pore length of about 35 μm. As electrolyte, an aqueous hydrofluoric acid solution (10 wt.% HF) has been employed. After the anodization, the porous silicon matrices have been dried in ambient air for about 24 h. Then, the magnetite nanoparticles have been infiltrated into the pores of these specimens. The used iron oxide particles of 3.8, 5 and 8 nm size have been fabricated by high-temperature decomposition [10], and they have been covered by an oleic acid shell of about 2 nm to prevent agglomeration and to stabilize them against oxidation. To vary the size of the nanoparticles, the organic solvent has to be modified. An increase of the boiling temperature of the solvent results in an increase of the particle size [11]. As usual, the particle size and its distribution have been determined by TEM [12]. While the infiltration of the particles into the pores of the porous silicon matrices has been performed, the temperature has been kept constant at 20°C for about 25 min. To facilitate the infiltration process, a magnetic field of 1 T has been applied perpendicular to the sample surface. To characterize the infiltrated particles inside the pores, scanning electron microscopy (SEM), together with energy dispersive X-ray (EDX) spectroscopy and EDX mapping, has been mainly used. EDX maps show an almost homogeneous distribution of iron and oxygen over the whole porous layer [12]. Figure 1 shows a SEM image of a cross-sectional region of a specimen containing Fe_3_O_4_ nanoparticles with a mean diameter of 8 nm within the pores. A verification of the appearance of the nanoparticles in the Fe_3_O_4_ phase has been carried out by X-ray diffractometry showing an inverse spinel structure with a lattice parameter of 8.38 (JCPDS 19–629) [13].

The magnetic properties of the nanocomposite system have been investigated by SQUID-magnetometry (S600, Cryogenic Ltd., London, UK) and with a vibrating sample magnetometer (PPMS, Quantum Design Inc., San Diego, CA, USA). During these measurements, the temperature has been varied from 4 to 300 K to gain zero-field-cooled (ZFC)/field-cooled (FC) magnetization curves. For field-dependent magnetization measurements, a magnetic field within ±6 T has been applied.

## Results and discussion

**Figure 2:**
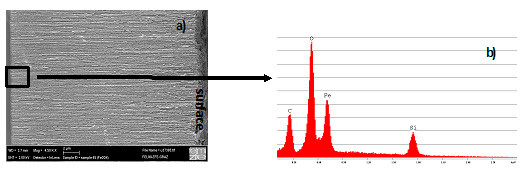
**Assessment of iron oxide particles within porous silicon. (a)** Scanning electron micrograph of a cross-sectional region of a porous silicon sample with infiltrated magnetite nanoparticles with a size of 8 nm. **(b)** EDX spectrum taken at the marked region at the pore tips.

Mesoporous silicon samples have been infiltrated with Fe_3_O_4_ nanoparticles 8 nm in size and with different concentrations of the particle solution (8, 4 and 2 mg Fe/ml). Also, particles of different sizes (3.8, 5 and 8 nm) have been filled into the pores. Figure 2a shows the SEM image of a cross section of a specimen, and Figure 2b shows the EDX spectrum gained from the marked region near the pore tips. EDX mapping of the samples shows that the particles are homogeneously distributed over the entire porous layers [12]. The magnetic behavior of the achieved nanocomposites has been examined with regard to the magnetic interactions between the particles within the pores. Dependent not only on the size but also on the distance between the particles, which has been adjusted by various concentrations of the particle solution used for pore filling, the blocking temperature *T*_B_, which indicates the transition between superparamagnetic behavior and blocked state, has been shifted. By varying the particle size but using the same concentration (8 mg Fe/ml) of the particle solution for different samples, ZFC/FC measurements show that the blocking temperature decreases from *T*_B_ = 170 K (8-nm particles) to *T*_B_ = 15 K (5-nm particles) and further to *T*_B_ = 10 K (3.8-nm particles). Field-dependent magnetization curves offer a coercivity of about 370 Oe below *T*_B_ (*T* = 4 K), and above *T*_B_, the coercivity nearly vanishes (*H*_C_ ~ 10 Oe).

**Figure 3:**
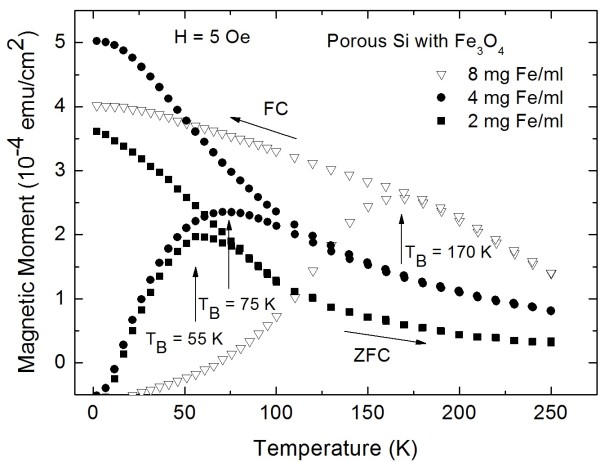
**Shift of *T***_**B**_** towards a lower temperature.** Varying the magnetite solution from 8 to 4 mg Fe/ml and further to 2 mg Fe/ml leads to a shift of the blocking temperature from *T*_B_ = 170 K (triangles) to *T*_B_ = 75 K (circles) and to *T*_B_ = 55 K (squares). For all three solutions, the size of the particles is 8 nm.

A decrease of the concentration of the particle solution results in a decrease of the magnetic coupling between the particles, which leads to a shift of the blocking temperature to lower values. Figure 3 shows the shift of *T*_B_ towards a lower temperature (*T*_B_ = 170 to 55 K) with increasing distance between the iron oxide particles with a diameter of 8 nm by changing the concentration of the Fe_3_O_4_ solution from 8 to 2 mg Fe/ml.

**Figure 4:**
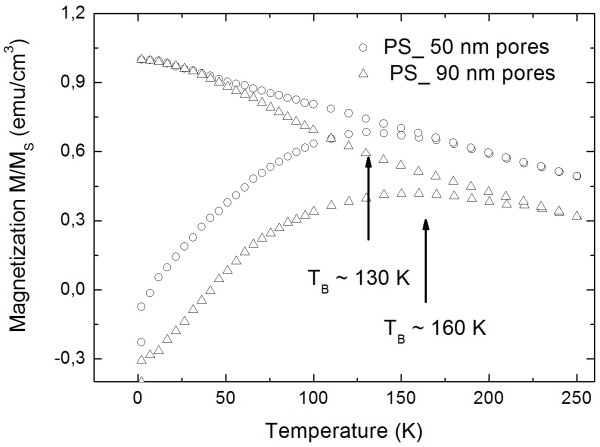
**Temperature-dependent magnetization of magnetite-filled porous silicon, whereas the porous silicon matrices exhibit different morphologies.** The particle size used was 8 nm in diameter. The lower blocking temperature (*T*_B_ ~ 130 K) results from nanoparticles within pores with an average diameter of 50 nm and a concomitant mean pore distance of 60 nm. The higher *T*_B_ ~ 160 K has been measured on a sample with pore diameters of about 90 nm and pore distances of about 35 nm.

Furthermore, the modification of the porous silicon template results also in a change of the transition temperature due to the variation of the particle distance of adjacent pores. The thickness of the silicon skeleton increases with decreasing pore diameter. Figure 4 shows the shift of *T*_B_ to a lower temperature, from 160 to 130 K, with decreasing pore diameter (from 90 to 50 nm) and increasing distance between the pores (from 35 to 50 nm) in the porous silicon matrix. An estimation of the amount of iron oxide particles infiltrated within the porous silicon matrix has been carried out [14] by taking the magnetic moment of one iron oxide particle of 1.49 × 10^−18^ emu, which results in about 1.3 × 10^19^ particles/cm^3^.

The variation of the distance between the particles within the pores or between adjacent pores results in a modification of the strength of the dipolar coupling among the iron oxide nanoparticles. In general, one can say that the blocking temperature of the system depends on the particle size on the one hand. On the other hand, for a constant particle size, the *T*_B_ can be modified by changing the distance between the particles within the pores, and a dramatic shift can be observed (see Figure 3). Also, in the case of varying the morphology of the porous silicon matrices, the *T*_B_ can be modified in a smaller regime (see Figure 4) due to the change of the distance between the pores.

The blocking temperature is a crucial factor that has to be addressed in the case of biomedical applications because of the importance of the superparamagnetic behavior of the particles and the porous silicon/iron oxide composite system. Above T_B_, the nanoparticles do not magnetically interact due to the randomization of their magnetization, whereas below T_B_, the particles do interact and offer a ferromagnetic-like behavior. To facilitate the utilization of the particles in biomedicine, the blocking temperature has to be below room temperature to guarantee the disappearance of the remanence when the magnetic field is switched off.

## Conclusions

In the frame of this work, the magnetic properties of Fe_3_O_4_ nanoparticles infiltrated within the pores of porous silicon have been investigated with respect to the transition between superparamagnetic behavior and blocked state. To fabricate distinct composite systems suitable, e.g., for magnetic field-guided drug delivery, the porous silicon template, as well as the loading of the pores with the particles, has to be adjusted. The nanocomposite should be superparamagnetic at room temperature but also offer a magnetic moment as big as possible. To ensure that there is no remanence after the external field has been switched off, magnetic coupling between the SPM particles has to be sufficiently low. A low coupling is in competition with the particle size and the maximized magnetic moment of individual particles, respectively, which means that a decrease of the size of the core particle results not only in weaker coupling but also in lower magnetic moments. The blocking temperature is decreased due to less coupling between smaller particles whereas the minimum distance is constant due to the same organic coating of about 2 nm in thickness for all particle sizes. To achieve systems with distinct magnetic properties, a variation of the particle size, as well as of the concentration of the particle solution for the pore filling, is appropriate.

## Competing interests

The authors declare that they have no competing interests.

## Authors’ contributions

PG performed the sample preparation by anodization as well as the infiltration of the iron oxide nanoparticles into the templates. PG and KR carried out the magnetization measurements by SQUID and VSM. PM prepared the iron oxide nanoparticles. PP carried out the SEM investigations. MR assisted in performing the VSM measurements. All authors read and approved the final manuscript.
